# Synergistic Epidemic or Syndemic: An Emerging Pattern of Human Diseases

**DOI:** 10.7759/cureus.48286

**Published:** 2023-11-05

**Authors:** Aditi Shelke, Saurabh Shelke, Sourya Acharya, Samarth Shukla

**Affiliations:** 1 Department of Medicine, Jawaharlal Nehru Medical College, Datta Meghe Institute of Higher Education and Research, Wardha, IND; 2 Department of Pathology, Jawaharlal Nehru Medical College, Datta Meghe Institute of Higher Education and Research, Wardha, IND

**Keywords:** non-communicable disease, hiv, sava syndemic, covid-19, synergistic epidemic, syndemic

## Abstract

Synergistic epidemics refer to the phenomenon where the occurrence and interaction of multiple diseases or health conditions exacerbate their individual impact, leading to complex health challenges and increased vulnerability in populations. Syndemics are a complex, multilevel phenomenon. In a population with biological interactions, a syndemic is the accumulation of two or more concurrent or sequential epidemics, which significantly worsens the situation. Disease concentration, disease interaction, and their underlying social forces, such as poverty and social inequality, are the fundamental concepts. Extensive political, economic, and cultural factors have contributed to cluster epidemics of several infectious diseases, particularly HIV and tuberculosis. Concerning the SAVA (substance abuse, violence, AIDS) syndemic, this narrative review article explores the complex interactions between substance abuse, violence, and HIV/AIDS. Further, it describes in-depth interactions between the COVID-19 syndemic's health conditions, societal factors, biological factors, and global dynamics. The review also emphasizes how infectious and non-communicable diseases interact, emphasizing how having one condition can make the severity and outcomes of another worse. It investigates the causes of synergistic epidemics and the impact of environmental factors. Syndemics acknowledge that the presence of one condition can worsen the severity and progression of others and take into account the intricate relationships between diseases. We can create more efficient plans to enhance health outcomes, lessen disparities, and promote healthier communities by understanding the connections between disorders and the underlying social determinants. This narrative review provides insights into the emerging patterns of human diseases within synergistic epidemics.

## Introduction and background

In recent decades, the global health landscape has witnessed a profound shift, with the emergence of synergistic epidemics that present novel challenges for public health [1]. The syndemic framework describes the synergistic interactions between two or more concurrent epidemics and the intricate structural and social dynamics that support them, which result in an excess of disease burden. "Syndemic" was a term coined by Merrill Singer to describe how AIDS, violence, and drug abuse are interconnected [2]. The existence of two or more disease states that interact poorly, adversely impact one another's disease trajectories, increase vulnerability, and are made more harmful by historical injustices, is referred to as a syndemic. It provides a conceptual framework for comprehending social and economic and political and environmental conditions that have an impact on communities and are made worse by those environments. A syndemic, also referred to as a synergistic epidemic, is more than just a comorbidity, in other words. Merrill Singer, a medical anthropologist, first used the term "syndemic" to describe the connections between drug abuse, violence, and AIDS (SAVA), which had become a serious health epidemic in Hartford, Connecticut, USA, in the 1990s [3]. The idea of a syndemic is based on three concepts: disease concentration, disease interaction, and the large-scale societal dynamics that cause them. The theory of disease concentration states that unfavorable socioeconomic conditions lead to the co-occurrence of two or more epidemics in particular temporal or geographic contexts [4]. Clinical medicine is closely related to the relationships between the diseases, which are primarily biological but can also be social and psychological. It helps us better understand how policy initiatives promote health and healthcare, recognizing that the relationships and concentrations of disease have a history and perhaps a common genesis. This social context is also provided for clinical care [5]. The simultaneous presence of COVID-19 and existing epidemics like cancer, diabetes, and HIV-positive people with compromised immune systems are at an increased risk of illness and death [6]. As we navigate an era marked by the COVID-19 pandemic, the idea of synergistic epidemics has become more and more critical. The pandemic has highlighted the interconnectedness of diseases and flaws in our healthcare systems. It has demonstrated how severe outcomes and elevated mortality rates can result from the interaction between an active viral infection and preexisting non-communicable diseases. These lessons emphasize how crucial it is to investigate synergistic epidemics and create integrated strategies to protect global health [7]. Moreover, the pandemic has exposed the interconnectedness of human health and the environment. Environmental factors, such as air pollution and climate change, can exacerbate disease interactions and amplify their impact. For instance, according to studies, air pollution can increase the susceptibility to respiratory infections, including COVID-19, while climate change contributes to the spread of vector-borne diseases [8].

Methodology

**Figure 1:**
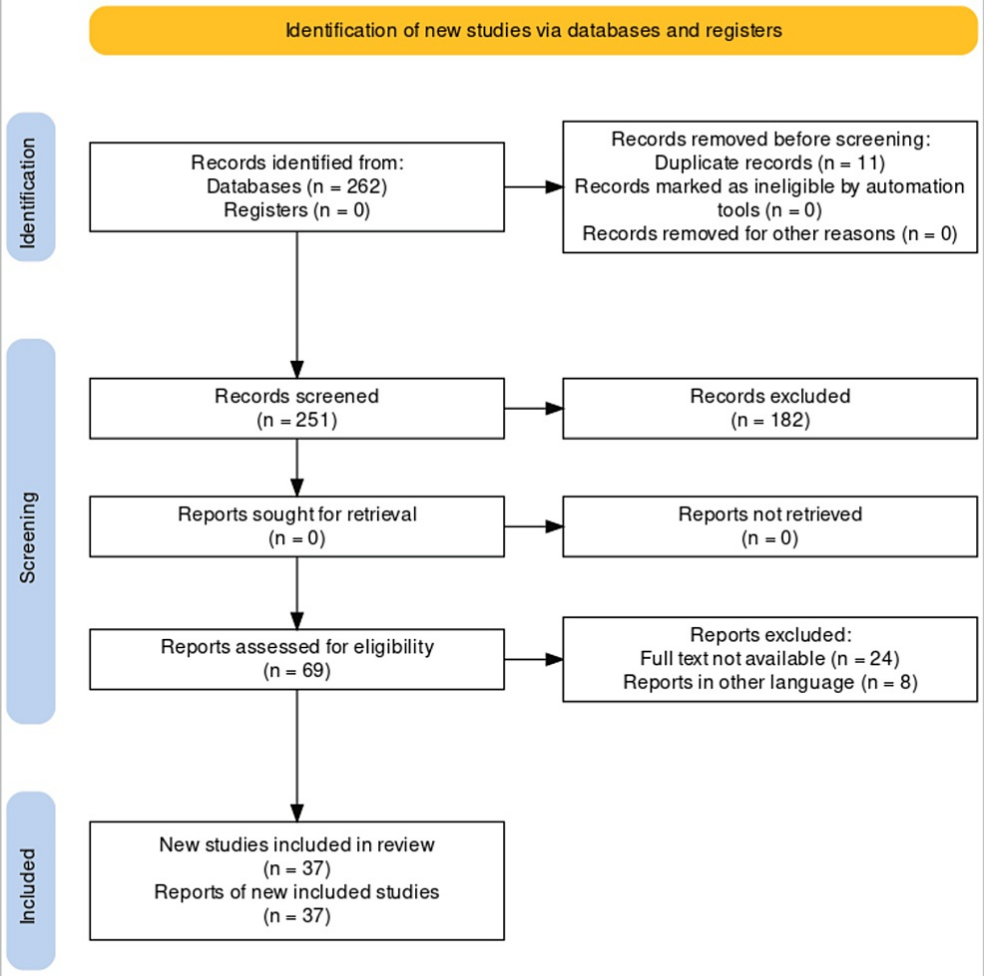
PRISMA flow diagram of synergistic epidemics or syndemics Adopted from the Preferred Reporting Items for Systemic Reviews and Meta-analyses (PRISMA)

A comprehensive literature search in English was conducted using electronic databases PubMed and Google Scholar in June 2023. The search terms were “syndemic”, “SAVA,” “COVID-19,” “synergistic epidemic,” ((Syndemic) AND (SAVA) AND (COVID-19) AND (Synergistic epidemic) AND (Non communicable disease)). Some of the content of this article has been taken from the key references of their respective bibliographies and citations. The inclusion criteria comprised all the articles that were published in the last ten years that discussed synergistic epidemics for which PubMed or the publisher provided open access. The articles that were excluded were not retrievable and discussed either because of being written in some other language or as the full text was not available. A total of 262 articles were found, and, out of which, 37 were chosen to be included because it was determined that they were pertinent. They were selected following Preferred Reporting Items for Systemic Reviews and Meta-Analysis (PRISMA) guidelines. A comprehensive outline of the selection method is given in Figure 1.

## Review

According to the syndemics theory, it is crucial to understand how epidemic synergies work to comprehend what diseases arise, where they occur, and why. A syndemic is characterized by three main traits: Two or more epidemics must (1) co-occur in specific contexts; (2) interact meaningfully and frequently through biological processes but occasionally through social or psychological processes; and (3) share one or more upstream factors that are responsible for their interaction and co-occurrence. These dynamics may be structural, social, cultural, ecological, or economic [5].

Syndemic theory brings together a variety of disciplines to pinpoint, define, and tactically address the complex burdens of numerous diseases affecting vulnerable populations. This system makes it easier to advance healthcare and uphold human rights. Syndemic theory enables us to pinpoint biological links between illnesses that are present simultaneously, which may conceal genuine associations between two or more conditions. It describes the conditions under which various health issues interact, suggests potential interventions, and aims to address connected social and health issues with the intention of reducing their combined effects [9].

The phrases "comorbidity" and "syndemic" are distinct as the first term frequently exclusively refers to nosography, while the latter term often relates to concurrent socioeconomic issues (Table 1). However, there are some similarities between the two names. Without or with the influence of social or economic factors, two or more diseases may coexist. Conversely, the term "syndemic" describes the combined effects of additional economic and social factors (e.g., poverty and exploitation), which make someone more vulnerable to disease [10]. The term "syndemogenesis" refers to all methods, stages, and interactions between diseases and societal situations that result in the emergence of syndemics [11].

**Table 1:** Differences between syndemic, endemic, and pandemic Differences between syndemic, endemic, and pandemic are mentioned above [12]

SYNDEMIC	ENDEMIC	PANDEMIC
SYN (COMBINED)+ DEMOS	EN (WITHIN) + DEMOS	PAN (ALL) + DEMOS
Occurrence of two or more epidemics at a single time	A disease that affects a society or a country at a roughly constant rate	An epidemic that is actively spreading to multiple regions across the globe
The development of two or more epidemics in a population, either concurrently or sequentially, with biological interactions	A disease that is constantly maintained at a baseline level in a limited geographic area without any transfer of infection taking place from the outside	A disease affecting large populations across borders
SAVA syndemic	Chickenpox is an endemic in the UK	COVID-19

SAVA syndemic

The SAVA syndemic, which combines the epidemics of substance abuse, violence, and HIV/AIDS, is especially prevalent among urban poor women and is possibly linked to unfavorable HIV outcomes. In the context of the SAVA syndemic, substance abuse, violence, and HIV/AIDS are closely intertwined, with each factor influencing and amplifying the others.

Substance Abuse

Substance abuse, including the abuse of drugs and alcohol, is a serious public health issue. Substance abuse can impair judgment, increase risky behaviors, and compromise overall health, making individuals more vulnerable to violence and HIV/AIDS transmission. Both alcohol and drug abuse decrease compliance to antiretroviral therapy for HIV and cause viral suppression to fail [13].

Violence

Violence, including interpersonal violence, domestic violence, and community violence, has a detrimental impact on individuals and communities. Violence contributes to physical and psychological harm, disrupts social support systems, and hampers access to healthcare services, thereby increasing the risk of substance abuse and HIV/AIDS transmission.

*HIV/AIDS* 

Human immunodeficiency virus (HIV) infection and acquired immunodeficiency syndrome (AIDS) are global health challenges. Because of factors like widened sexual networks, concurrent relationships, increased substance abuse, increased prevalence of the virus in the population, disruptions in social dynamics, and increased participation in casual and commercial sexual activities, HIV transmission tends to be elevated among rural-to-urban and seasonal migrants [14].

The SAVA syndemic recognizes that drug abuse and violence can increase the risk of HIV transmission and impede efforts to prevent and treat HIV. For example, substance abuse may lead to risky sexual behaviors, while violence can limit individuals' ability to negotiate safe sex practices or access healthcare [15].

**Figure 2:**
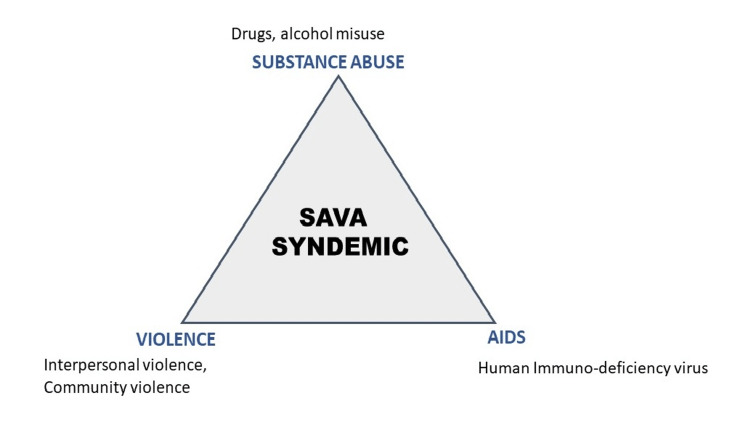
SAVA syndemic The triad of SAVA syndemic (AIDS, substance abuse, and violence) Image credits: Aditi Shelke

Figure 2 shows the SAVA syndemic triad.

Asthma and synergistic association

Asthma is an intriguing area for conceptualizing the syndemic idea, ranking seventh in prevalence among chronic illnesses in the United States and being a significant source of serious illness in children. People with asthma have been reported to have higher rates of coinfection with several viruses, such as the influenza A virus and the respiratory syncytial virus (RSV). Furthermore, a larger likelihood of asthma exacerbation and more severe triggered episodes are more likely in newborns and young children with asthma and RSV infection, raising the possibility of a synergistic effect [16]. 

COVID-19 syndemic association

The syndemic perspective in the case of COVID-19 recognizes that it is not just a health crisis but also intersects with several other factors, including socioeconomic disparities, mental health problems, and systemic inequalities. These interconnected issues exacerbate the overall impact of COVID-19 and contribute to its disproportionate effects on vulnerable populations.

The COVID-19 syndemic can be understood through various dimensions:

Health and Disease

COVID-19 interacts with preexisting health conditions, such as respiratory illnesses, cardiovascular diseases, and diabetes, leading to worse outcomes and increased mortality rates among those with comorbidities. Patients with non-communicable diseases like type 2 diabetes, hypertension, and ischaemic heart disease account for the majority of COVID-19 hospital deaths [17]. The stress on healthcare systems and resources also affects the overall management of various health conditions, leading to delayed or inadequate care for non-COVID-19-related illnesses [18].

Socioeconomic Factors

The pandemic has brought attention to and widened socioeconomic divides already present. Marginalized communities, low-income people, and critical workers were disproportionately impacted by COVID-19's effects, which included job losses, economic instability, and disruptions in education. These differences also have an impact on access to healthcare services and health outcomes [19].

Social and Behavioral Factors

The interconnectedness of individual behaviors and societal reactions to the pandemic is another aspect of the COVID-19 syndemic. Vaccination rates, disease transmission rates, and outcomes vary depending on the population's compliance with public health measures like mask use, social withdrawal, and vaccination [20].

Mental Health

**Figure 3:**
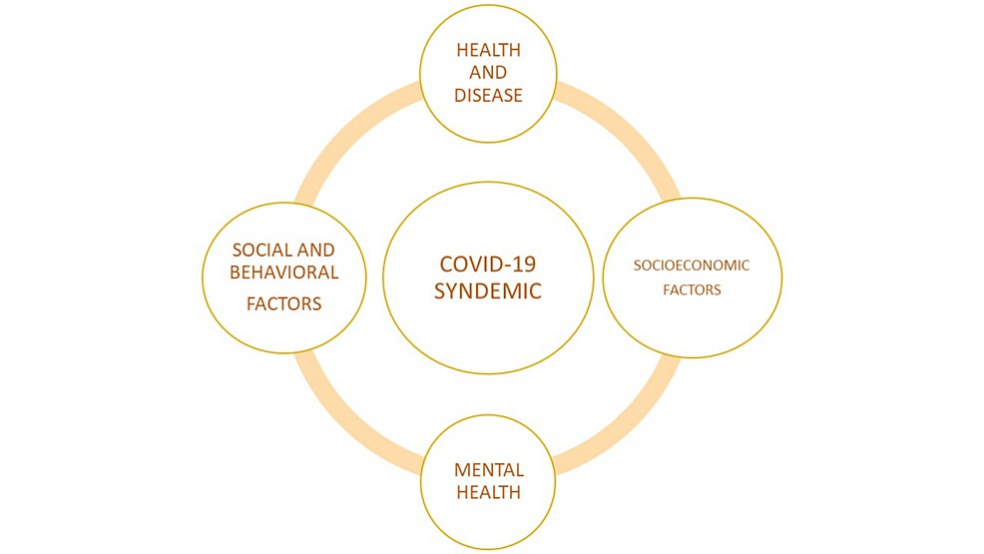
Dimensions of the COVID-19 syndemic Complex interplay between the COVID-19 syndemic, health and disease, socioeconomic factors, mental health, and social and behavioral factors Image credits: Aditi Shelke

Anxiety, depression, and stress-related disorders have all increased in frequency because of the COVID-19 pandemic. Along with the effects of COVID-19 on physical health, the isolation, grief, and uncertainty brought on by the pandemic, as well as its social and economic repercussions, have contributed to a syndemic of mental health problems. Among other clinical and substance use disorders, the COVID-19 pandemic is negatively affecting the minds of people causing anxiety, depression, psychosis, and suicidal ideation both immediately and over time [21]. Anxiety, post-traumatic stress disorder, depression, and sleep disorders can affect people working in the healthcare industry, especially frontline and migrant employees and those who interact with the general public. Meanwhile, the prospect of losing one's job, remaining alone for a long time, and facing an uncertain future causes negative emotions, especially in younger people and those with higher levels of education [22]. Figure 3 shows the dimensions of COVID-19 syndemic association.

India experienced an increasing level of cases of mucormycosis, a lethal fungal infection that is also known as black fungus, during the second wave of COVID-19. With a fatality rate of 50%, the Indian government declared it an epidemic because of its novel emergence [23].

VIDDA syndemic

Mendenhall identified the VIDDA syndemic among first- and second-generation Mexican immigrants who lived in Chicago. It consists of violence, immigration, depression, diabetes, and abuse (VIDDA: violence, immigration, depression, diabetes, abuse). It is a classic example of syndemics that illustrates the "pathological reality and social aspects" of syndemics. According to academics, diabetes and depression both tend to make life more stressful and add to each other's burdens within the VIDDA syndemic. According to epidemiological studies, diabetic patients have a two times higher prevalence of depression than people in other categories [24]. Another classic example of a supersyndemic condition is metabolic syndrome. Although it is defined by certain conditions like obesity, diabetes, insulin resistance, hypertension, or increased triglycerides, the issue is caused by a number of other factors like the use of fossil fuels as the primary energy source rather than human muscle, urbanization, social inequalities, and the emergence of global warming, which all combined to cause the issue [21].

Association between infectious and non-communicable diseases 

The interactions between infectious and non-communicable diseases are significant contributors to the development and impact of syndemics. The interplay between infectious and non-communicable diseases within a syndemic framework is characterized by complex interactions and shared risk factors. Several key aspects can be observed in understanding these interactions:

Common risk factors: Infectious diseases and non-communicable diseases frequently have similar risk factors. For instance, both infectious diseases (like respiratory infections) and non-communicable diseases (like cardiovascular diseases, diabetes, and sedentary behavior) can be influenced by lifestyle factors such as sedentary behavior, poor nutrition, and tobacco use. These common risk factors provide a common environment for the emergence and spread of numerous diseases [25].

Bidirectional relationships: The risk and severity of non-communicable diseases can be exacerbated by infectious diseases, and vice versa. For instance, people with weakened immune systems brought on by HIV/AIDS are more vulnerable to non-communicable conditions like cardiovascular disease and some cancers. Similarly, some non-communicable diseases, such as obesity and diabetes, can increase the susceptibility to infections and impede their treatment and management [26].

Health system challenges: Healthcare systems in rural areas face particular difficulties when infectious and non-communicable disease syndemics occur. There is a need to determine the healthcare delivery gaps to effectively prevent, diagnose, and manage these [27]. 

Disproportionate impact: Disadvantaged populations from low socioeconomic backgrounds have a higher rate of falling ill and less access to healthcare and fail to meet medicine adherence, exacerbating health disparities [28].

A thorough and integrated strategy is needed to address the interactions between infectious and non-communicable diseases within the syndemic framework. This covers coordinated efforts for both infectious and non-communicable disease prevention, early detection, treatment, and management. To lessen the effects of syndemics and decrease health disparities, it is also essential to address shared risk factors and social determinants of health [29].

A syndemic interacts with a variety of comorbid medical diseases like obesity and diabetes as well as social, political, and ecological elements to exacerbate existing non-communicable diseases. COVID-19 is regarded as a syndemic by people living with non-communicable diseases (PLWNCD). Studies have shown that PLWNCD in developing countries have greater rates of frailty, malnutrition, psychosocial issues, and coinfections, including pathogens with antibiotic resistance. Morbidity and death rise when COVID-19 is combined with preexisting non-communicable diseases. Non-communicable diseases may have several traits with infectious symptoms, such as a proinflammatory state and a weakened innate immune response [30]. For instance, diabetes and tuberculosis (TB) together exemplify the synergistic relationship between an infectious disease and an NCD. Studies have shown that individuals with diabetes are more susceptible to TB infection and have poorer treatment outcomes, highlighting the need for integrated approaches to manage these co-occurring conditions [31].

Association with environmental factors

Synergistic epidemics are shaped by environmental factors, which significantly impact how diseases interact and have an amplified effect. Numerous environmental factors, including urbanization, climate change, and others, have been linked to the emergence and spread of various diseases. For instance, research into how air pollution affects respiratory infections has shown that areas with poor air quality have a higher risk of infectious diseases like COVID-19 [32]. Additionally, the expansion of vector-borne illnesses like malaria and dengue fever is influenced by the changing climate, highlighting the need for comprehensive strategies to address how environmental factors and disease emergence are interconnected [33].

Societal and behavioral determinants

The patterns and outcomes of synergistic epidemics are significantly influenced by social and behavioral determinants. Health disparities can be exacerbated and shaped by socioeconomic differences, lifestyle decisions, and cultural practices. The need for targeted interventions to address the social determinants of health is highlighted by studies showing, for instance, that people from low socioeconomic backgrounds are disproportionately affected by synergistic epidemics [34]. Further highlighting the complexity of disease interactions and the significance of a comprehensive approach to disease prevention and management, cultural practices and behaviors, including dietary practices and levels of physical activity, can have an impact on the prevalence and progression of both infectious diseases and non-communicable diseases [35]. The interactions between diseases have a noteworthy impact on the development and outcomes of diseases. Still, it has become increasingly clear as medical knowledge has increased that diseases do not always coexist with other illnesses and conditions and that the social context in which disease sufferers live is crucial to understanding how diseases affect people's health individually and collectively. Many times, rather than existing as separate conditions, multiple life-threatening diseases are concentrated in specific populations [36]. Policymakers and program implementers may find the theory of syndemics useful in their efforts to promote population health. According to the theory, syndemics are intricate, multilevel phenomena, and there are still plenty of opportunities to research how epidemics interact at the population and individual levels as well as how they shift across time and geography [37]. Table 2 represents a summary of the references.

**Table 2:** Summary of the references NCD: Non-communicable disease; DM: Diabetes mellitus; PTSD: Post-traumatic stress disorder

Authors	Year	Findings
Nakatani et al. [1]	2020	The emergence of new infectious diseases and antimicrobial resistance is a global concern. COVID-19 led to a focus on research and development and highlighted the significance of global cooperation and fair access to healthcare.
Shrestha et al. [2]	2020	Spatial epidemiology uses geographic information systems and statistics to understand the geographical landscape and environmental factors within which disease epidemic occurs.
Lancet [3]	2017	A syndemic is characterized by the coexistence of two or more diseases that negatively interact with one another, affecting the mutual course of a disease and enhancing vulnerability and exacerbating preexisting disparities.
Tsai et al. [4]	2017	The concept of syndemic is based on three ideas: disease concentration, disease interaction, and the large-scale social dynamics that cause them.
Mendenhall [5]	2022	The syndemic framework suggests that multiple factors collaborate synergistically and the population with the highest morbidity and mortality are most profoundly affected by these interactions.
Islam et al. [6]	2021	In some countries, there is a simultaneous occurrence of COVID-19 and chronic diseases alongside social disadvantage.
Zhu et al. [7]	2020	Lung tissue identification, antibody detection, and animal experiments suggested the novel coronavirus’s role in causing severe pneumonia in the outbreak of COVID-19.
Carracedo-Martínez Eduardo et al. [8]	2010	Comorbidity analysis, which focuses on hospital admissions, employs symmetric and time-stratified designs in the context of air pollution and health.
Mendenhall et al. [9]	2017	The application of syndemic theory to comorbidities in low- and middle-income countries is examined with an example of diabetes, HIV, tuberculosis, and depression.
Jakovljevic et al. [10]	2021	Comorbidity is the coexistence of several medical conditions in one person, whether they are independent, dependent on one another, or unrelated, often sharing factors like genetics. COVID-19 affects many organic systems such as the respiratory, cardiovascular, gastrointestinal, central nervous systems; the liver; the spleen; and the kidneys because of comorbidities.
Lerman [11]	2018	Depression is an example of syndemogenesis and interacts on both the structural and biological levels with other health conditions indicating the presence of multiple syndemics.
Sullivan et al. [13]	2015	Five social factors such as substance abuse, binge drinking, intimate partner violence, poor mental health, and sexual risk-taking have a clustering and an additive effect contributing to HIV suppression, supporting the syndemic effect.
Talman et al. [14]	2013	The interplay between climate change, environmental changes, HIV, pollution, and economic policies impact livelihoods, reliance on natural resources, and community well-being affecting both the people and the ecosystem.
Meyer et al. [15]	2011	The SAVA syndemic significantly affects woman’s lives and decision-making, particularly in vulnerable populations such as urban women, women of color, and criminals.
Zhao et al. [16]	2002	Respiratory syncytial virus-infected children have a higher level of eosinophils in their respiratory tract secretions, indicating a link between eosinophil infiltration and asthma exacerbation.
Kluge et al. [17]	2020	The prevention and control of NCDs are critical during COVID-19. However, measures like the implementation of lockdowns and social distancing negatively impact patients with NCDs by limiting healthcare resources.
Ebinger et al. [18]	2020	In our healthcare system, the severity of COVID-19 is influenced by age, gender, race, and comorbidities such that patients who are older, male, African-American, obese, and diabetic require a higher level of care.
Calcaterra [19]	2022	COVID-19 has revealed that people with NCDs and vulnerable populations like low-income countries, refugees, migrants, and minority groups face healthcare disparities. Cultural understanding is crucial for crisis response, and a new syndemic approach is needed to prevent future suffering.
Lahiri et al. [20]	2021	The socioeconomic crisis during COVID-19 affected the business, Indian households, and students severely.
Saqib et al. [21]	2023	Mental health, COVID-19, and mortality are interconnected and influenced by factors such as the evidence of multi-organ failure and syndemic interaction with NCDs.
Giorgi et al. [22]	2020	Organizational and employment factors affect mental health at the workplace, mainly affecting healthcare workers by increasing the risk of anxiety, depression, PTSD, and sleep disorders.
Rocha et al. [23]	2021	Syndemic association caused a rise in cases of mucormycosis during COVID-19.
Hayran et al. [24]	2019	Essential features of the syndemic are clustering, interaction, and an increase in the burden of disease. VIDDA syndemic, chronic kidney disease and tuberculosis syndemic, childhood anemia, and stunting syndemics are described.
Peters et al. [25]	2019	A healthy diet and physical activity decrease the risk of NCDs, while smoking, hypertension, and obesity increase the risk of NCDs to varying degrees.
Coates et al. [26]	2020	Infectious cases contribute to over 10% of the NCD burden, which surpasses the impact of common risk factors like dietary risks, tobacco use, and alcohol use.
Nath et al. [27]	2021	Recent data point to a shift towards NCDs in rural India because of behavioral risk, low awareness, and healthcare accessibility issues. Workforce shortages, insufficient medication, and poor quality of care highlight the need for improvements such as task shifting, mobile health technologies, and community engagement to counteract the NCD burden.
Basu [28]	2020	Addressing NCDs in resource-limited settings creates ethical dilemmas, exacerbated by COVID-19; possible solutions include extended drug supply, government medicine networks, and specialized care to promote patient wellbeing.
Tsai et al. [29]	2016	The article highlights the challenges with the common sum score approach in studying syndemics, stressing the need for better modeling and approaches to handle complex health interactions.
Yadav et al. [30]	2020	COVID-19 and NCDs have an interdependent relationship. COVID-19 enhances the risk factors associated with NCDs, while NCDs increase susceptibility to COVID-19.
Menon et al. [31]	2016	Aging and DM act synergistically to lower interferon-gamma, which increases susceptibility to tuberculosis, for which cell-mediated immunity is crucial.
Ali and Islam [32]	2020	Exposure to air pollution elevates the risk of infection and death from COVID-19 and impacts disease transmission and worsens the prognosis of COVID-19 patients.
Gutman et al. [33]	2020	Coinfection of malaria and COVID-19 cause more severe outcomes than either pathogen alone.
Mishra et al. [34]	2021	COVID-19 underscores the catastrophic effects for marginalized communities and emphasizes the need for sufficient support to survive the pandemic and future global threats.
Sharma et al. [35]	2023	Traditional dietary and physical activity practices are being displaced negatively due to delayed health system actions, limited knowledge, and environmental factors causing negative effects on health.
Singer et al. [36]	2006	Research states that relative poverty, social disadvantage, and chronic discrimination in healthcare access have a profound effect on individual and population health.
Tsai [37]	2018	Policymakers and program implementers can improve population health using the syndemic theory, which helps to explain the intricate interactions between epidemics at individual and population levels over time and space.

## Conclusions

The idea of synergistic epidemics and their new patterns in human diseases have been discussed in this narrative review article. The review has emphasized the complex relationships between infectious and non-communicable diseases and how social and environmental determinants of health shape these synergistic epidemics. Effective interventions cover the management of infectious and non-communicable diseases as well as their early detection, prevention, and control. The evidence emphasizes the pressing need for an all-encompassing and integrated strategy to deal with synergistic epidemics. The way that infectious diseases and drug misuse interact is a clear illustration of the wide-ranging effects of a syndemic. The syndemic is a result of the convergence of behavioral dynamics, structural inequities, and vulnerabilities rather than the result of a random convergence of epidemics. Greater infection rates, greater death rates, and long-lasting health disparities are the results of convergence. The COVID-19 pandemic has brought attention to long-standing disparities in healthcare delivery. Infection and mortality rates have historically been the highest among the world's poorest and most marginalized populations. Morbidity and mortality in COVID-19 are not exceptions. Reducing health inequalities, promoting health equity, and addressing the root causes of the spread of vulnerability should be the key goals of our efforts. Adopting a syndemic perspective demands a change in clinical strategy and encourages a broader perspective beyond specific illnesses.
